# Supplementary material for: Disulfiram/Copper Induces Antitumor Activity against Both Nasopharyngeal Cancer Cells and Cancer-Associated Fibroblasts through ROS/MAPK and Ferroptosis Pathways
Source: Cancers (Basel). 2020 Jan 6;12(1):138. doi: 10.3390/cancers12010138 (PMC7017005; doi:10.3390/cancers12010138)
Supplement: Supplementary file 1 [file cancers-12-00138-s001.zip › 5-8F-STR.pdf]

Cell Line Authentication Service

STR Profiling Report

Sample From: Sun Yat-sen University

Sample Type: Cell Line

Testing Method: STR Genotyping

Report Time: 6/6/2019

Add: Room 502, NO.1015 Longteng Rd , Songjiang District, Shanghai/ Tel:+86-021-33559491
Homepage: http://www.biowing.com.cn/ E-mail: market@biowing.com.cn

COMPANY STATEMENT

1. THIS REPORT IS ONLY RESPONSIBLE FOR THE SAMPLES ANALYZED.
2. THE TESTING RESULTS AND THE ORGANIZATION NAME WILL NOT BE USED FOR ADVERTISEMENT, COMMERCIAL EXHIBITIONS, COMMERCIAL PERFORMANCE AND OTHER COMMERCIAL ACTIVITIES.
3. OBJECTIONS SHOULD BE RAISED WITHIN FIFTEEN DAYS AFTER THE RECEIPT OF THIS REPORT.
4. THE PAPER REPORT WITH CONTENT ALTERING, ADDING OR WITHOUT THE STAMPED SEAL OF THE COMPANY ARE INVALID.

Testing Company: Shanghai Biowing Applied Biotechnology Co. Ltd

Address: Room 205, NO.885 Wangjiashe Road, Songjiang District, Shanghai

Tel: +86-021-33559491

Contact: Wenyao Zhang

E-mail:market@biowing.com.cn

Cell Line Authentication – STR Profiling Report

Sample code

Table 1. Sample Code

Customer's code	Company Code
5-8F	20190529-02

Sample Number :1

Sample Type: Cell line

Testing Type: STR

Testing Method:

DNA was extracted by a commercial kit from CORNING (AP-EMN-BL-GDNA-250G). The twenty STRs including Amelogenin locus were amplified by six multiplex PCR and separated on ABI 3730XL Genetic Analyzer. The signals were then analyzed by the software GeneMapper.

Data Interpretation:

Cell lines were authenticated using Short Tandem Repeat (STR) analysis asdescribed in 2012 in ANSI Standard (ASN-0002) by the ATCC Standards Development Organization (SDO) and in Capes-Davis et al., Match criteria for human cell line authentication: Where do we draw the line? Int J Cancer.2013;132(11):2510-9.

Add: Room 502, NO.1015 Longteng Rd , Songjiang District, Shanghai/ Tel:+86-021-33559491
Homepage: http://www.biowing.com.cn/ E-mail: market@biowing.com.cn

Test Results

1. STR profile

Table 2. STR and Amelogenin Genotyping Results of Cell line.

Loci	Sample information			Cell Bank information		
	Sample name：5-8F			control name：5-8F		
	**Allele1**	**Allele2**	**Allele3**	**Allele1**	**Allele2**	**Allele3**
D5S818	11	12		11	12	13
D13S317	10	12	13.3	10	12	13.3
D7S820	10	12		10	12	
D16S539	9	10		9	10	
VWA	14	16		14	16	17
TH01	7	9		6	7	9
AMEL	X	X		X	X	
TPOX	8	9	12	8	9	
CSF1PO	10	11		10	11	
D12S391	20	21		20	21	
FGA	21	21		18	21	
D2S1338	17	23		17	23	
D21S11	27	30		30	30	
D18S51	13	16		13	16	
D8S1179	12	12		12	12	
D3S1358	15	18		15	18	
D6S1043	11	14	18	11	14	18
PENTAE	17	17		17	20	
D19S433	13	13		13	13	
PENTAD	9	12		9	12	
D1S1656	12	15		12	15	

Add: Room 502, NO.1015 Longteng Rd , Songjiang District, Shanghai/ Tel:+86-021-33559491
Homepage: http://www.biowing.com.cn/ E-mail: market@biowing.com.cn

2. database annotation

Figure 1. STR matching analysis

**Cell line name**	5-8F
**Synonyms**	58F
**Accession**	CVCL_C528
**Resource Identification Initiative**	To cite this cell line use: 5-8F (RRID:CVCL_C528)
**Comments**	Omics: Deep RNAseq analysis.<br>Omics: Transcriptome analysis.
**Sequence variations**	CAPZA2-MET gene fusion (CelloPub=CLPUB00484).
**Disease**	Nasopharyngeal carcinoma (NCIt: C3871)
**Species of origin**	Homo sapiens (Human) (NCBI Taxonomy: 9606)
**Hierarchy**	Parent: CVCL_6946 (SUNE1)<br>Children:<br>CVCL_S665 (5-8F/Erbitux)
**Category**	Cancer cell line

Note: The STR online match analysis of the test cell against EXPASY database, showing cell number (Cell No.) and cell name.

3. Authentication

☑ The submitted sample profile is human, but not a match for any profile in the DSMZ STR database. As the STR database of 5-8F was not logged in, the matching result can not be displayed, but the sample is basic match with the control sample of **5-8F** . If the paper was to publish, these data could be submitted to the magazine

☐ The submitted profile is an exact match for the following human cell line(s) in the DSMZ STR database (8 core loci plus Amelogenin): / .

☐ The submitted profile is similar to the following DSMZ human cell line: /.

- **Note:** A cell line can be considered to be authenticated when 80% (exact match) of the alleles in its STR profile match profiles from tissue or other cell line samples from that donor or from database. Cell lines with between a 55% to 80% (similar) match require further profiling for investigation of relatedness.

Appendix

1. Genotyping Strategy and Site Distribution

Table S1. Experimental Strategy and Sites

	Strategy 1	Strategy 2	Strategy 3	Strategy 4
1	D3S1358	D8S1179	D19S433	AMEL
2	VWA	D21S11	TH01	D1S1656
3	D7S820	D16S539	D13S317	D5S818
4	CSF1PO	D2S1338	TPOX	D12S391
5	PENTAE	PENTAD	D18S51	FGA
6			D6S1043	

The allele match algorithm compares the 8 core loci plus amelogenin only, even though alleles from all lociwill be reported when available.

2. DSMZ tools was used to carry on the cell line comparison, which contains 2455 cell lines STR data from ATCC, DSMZ, JCRB ,ECACC， GNE and RIKEN databases. If the cell is not included in the above cell library, users need to compared with other databases.

Technician: Jianan Zhang

Checked by: Ning Qian

Issued by: Yang Bai

Issue date: 6/6/2019

Add: Room 502, NO.1015 Longteng Rd , Songjiang District, Shanghai/ Tel:+86-021-33559491
Homepage: http://www.biowing.com.cn/ E-mail: market@biowing.com.cn
