# Supplementary material for: Disulfiram/Copper Induces Antitumor Activity against Both Nasopharyngeal Cancer Cells and Cancer-Associated Fibroblasts through ROS/MAPK and Ferroptosis Pathways
Source: Cancers (Basel). 2020 Jan 6;12(1):138. doi: 10.3390/cancers12010138 (PMC7017005; doi:10.3390/cancers12010138)
Supplement: Supplementary file 1 [file cancers-12-00138-s001.zip › HSF-STR.pdf]

# Cell Line Authentication – STR Profiling

Sample from: FuHeng Cell Center，Shanghai，China

esting Method: STR Genotyping
Report Time:Jpr 27, 2018

Cell Line Authentication - STR Profiling Report

## Sample code

Table 1. Sample Code

| Customer's code | Company Code |
|---|---|
| 403 | 20180427-05 |

Sample Number:1

Sample Type: Cell line

Testing Type: STR

Sample From: FuHeng Cell Center，Shanghai，China

## Testing Method:

DNA was extracted by a commercial kit from CORNING (AP-EMN-BL-GDNA-250G). The

twenty STRs including Amelogenin locus were amplified by six multiplex PCR and separated on

ABI 3730XL Genetic Analyzer. The signals were then analyzed by the software GeneMapper.

## Data Interpretation:

Cell lines were authenticated using Short Tandem Repeat (STR) analysis as described in 2012 in

ANSI Standard (ASN-0002) by the ATCC Standards Development Organization (SDO) and in

Capes-Davis et al., Match criteria for human cell line authentication: Where do we draw the line?

Int J Cancer.2013;132(11):2510-9.

# Test Results:

## 1. Result

Table 2. Matching information on the cell lines

| Sample Code | Multi-allele | Cell line matched | Cell Bank | Percentage |
|---|---|---|---|---|
| 20180427-05 | NO | | DSMZ | |

Multi-allele means some STR contain more than two loci.

## 2. Sample Description

20180427-05 The DNA of the cell lines found to basic match the type of cell lines in a cell lineretrieval,CRC database shows that cells called HSF corresponding to the cell number 3153C0001000000173. No multiple alleles were found in this cell line.

## 3. Genotyping Result

| STR and Amelogenin Genotyping Results of Cell line 20180427-05 | | | | | | |
|---|---|---|---|---|---|---|
| Loci | **Sample**: 403 | | | **Cell Bank information**: HSF | | |
| | Allele1 | Allele2 | Allele3 | Allele1 | Allele2 | Allele3 |
| D5S818 | 9 | 11 | | | | |
| D13S317 | 11 | 12 | | | | |
| D7S820 | 12 | 13 | | | | |
| D16S539 | 9 | 10 | | | | |
| VWA | 14 | 16 | | | | |

| | | | | | | |
|---|---|---|---|---|---|---|
| TH01 | 7 | 9 | | | | |
| AMEL | X | Y | | X | Y | |
| TPOX | 8 | 11 | | | | |
| CSF1PO | 11 | 12 | | | | |
| D12S391 | 18 | 22 | | | | |
| FGA | 23 | 24 | | | | |
| D2S1338 | 24 | 25 | | | | |
| D21S11 | 29 | 31.2 | | | | |
| D18S51 | 18 | 19 | | | | |
| D8S1179 | 15 | 15 | | | | |
| D3S1358 | 17 | 17 | | | | |
| D6S1043 | 12 | 19 | | | | |
| PENTAE | 10 | 13 | | | | |
| D19S433 | 14 | 15.2 | | | | |
| PENTAD | 8 | 11 | | | | |

# Others

1. Genotyping Strategy and Site DistributionAttached Table. Experimental Strategy and Sites

| | **Strategy 1** | **Strategy 2** | **Strategy 3** | **Strategy 4** |
|---|---|---|---|---|
| 1 | TH01 | TPOX | D3S1358 | AMEL |

| | | | | |
|---|---|---|---|---|
| 2 | D12S391 | VWA | D13S317 | D5S818 |
| 3 | D7S820 | D8S1179 | D6S1043 | D2S1338 |
| 4 | CSF1PO | PENTAD | D16S539 | D21S11 |
| 5 | FGA | | D19S433 | D18S51 |
| 6 | PENTAE | | | |

The allele match algorithm compares the 8 core loci plus amelogenin only, even though alleles from all loci will be reported when available.

**2. STR database comparison**

DSMZ tools was used to carry on the cell line comparison, which contains 2455 cell lines STR data from ATCC, DSMZ, JCRB ,ECACC and RIKEN databases. If the cell is not included in the above cell library, users need to compared with other databases.

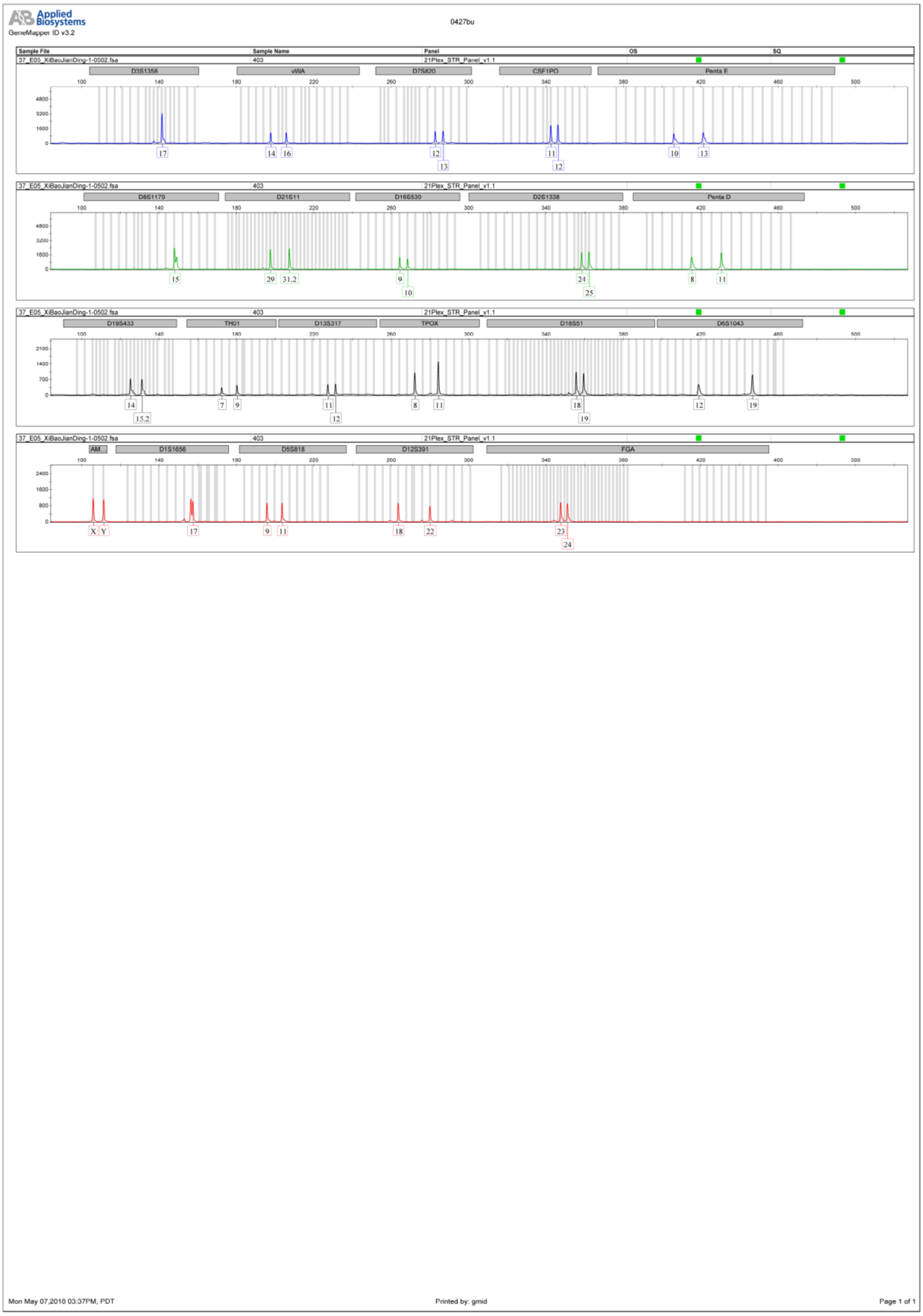


[redacted]签发日期：

2018 年 5 月
