# Supplementary material for: Disulfiram/Copper Induces Antitumor Activity against Both Nasopharyngeal Cancer Cells and Cancer-Associated Fibroblasts through ROS/MAPK and Ferroptosis Pathways
Source: Cancers (Basel). 2020 Jan 6;12(1):138. doi: 10.3390/cancers12010138 (PMC7017005; doi:10.3390/cancers12010138)
Supplement: Supplementary file 1 [file cancers-12-00138-s001.zip › Supplementary - Whole Western blot images.pdf]

# Supplementary Data

## Whole Western blot images

### Figure 2

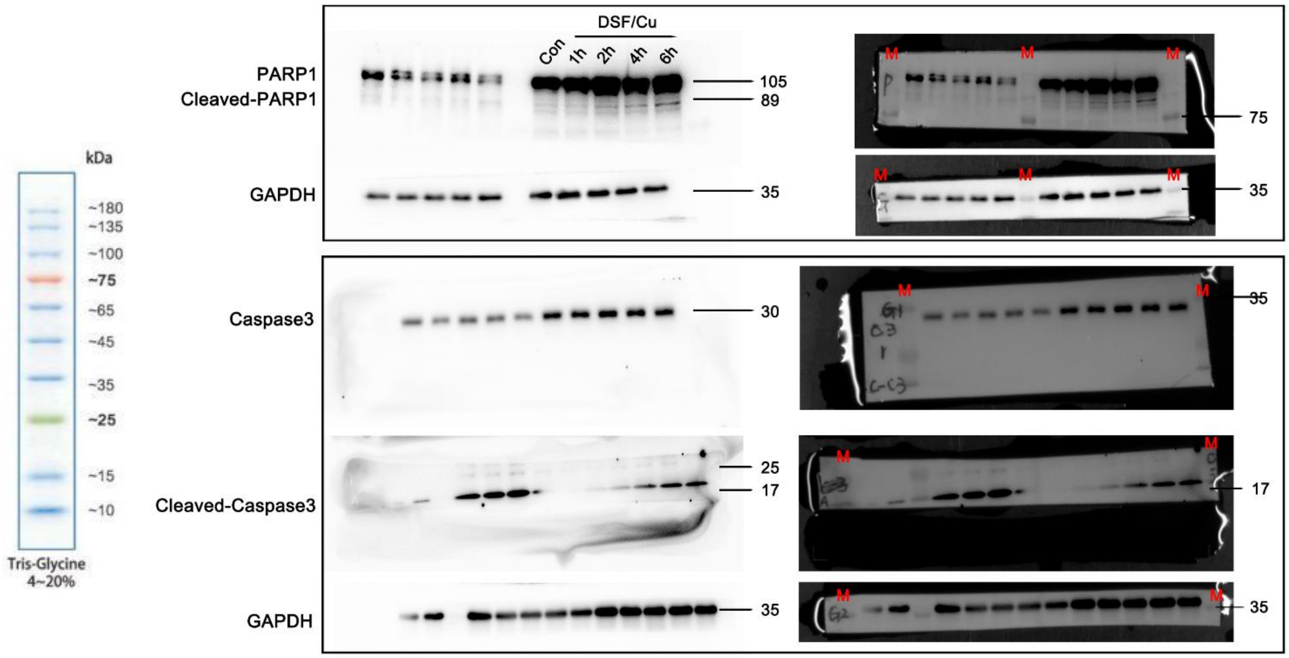

# Figure 3

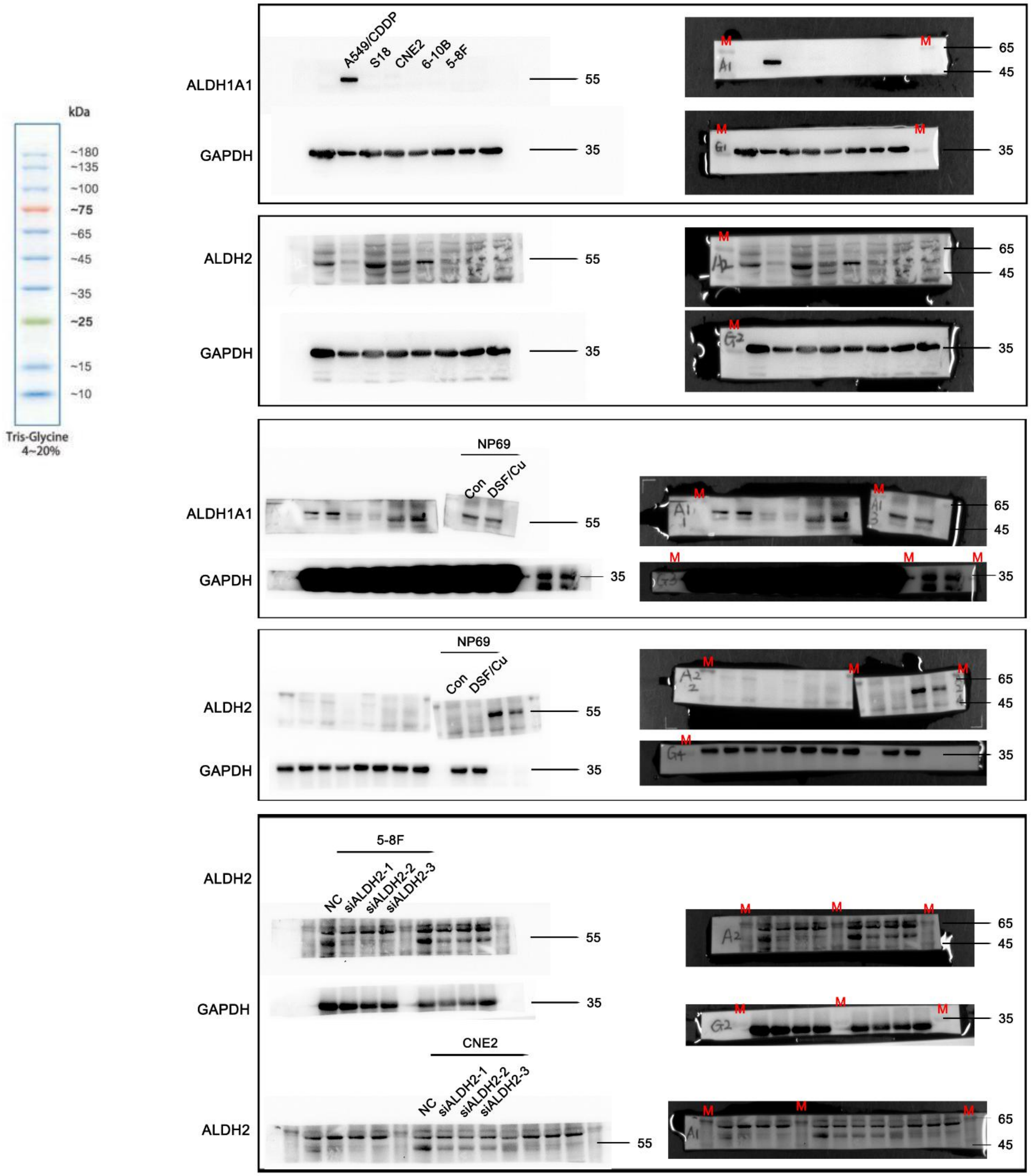

kDa
~180
~135
~100
~75
~65
~45
~35
~25
~15
~10
Tris-Glycine 4~20%
A549/CDDP
S18
CNE2
6-10B
5-8F
ALDH1A1
GAPDH
ALDH2
NP69
Con
DSF/Cu
5-8F
NC
siALDH2-1
siALDH2-2
siALDH2-3
CNE2
55
35
65
45
M

# Figure 4

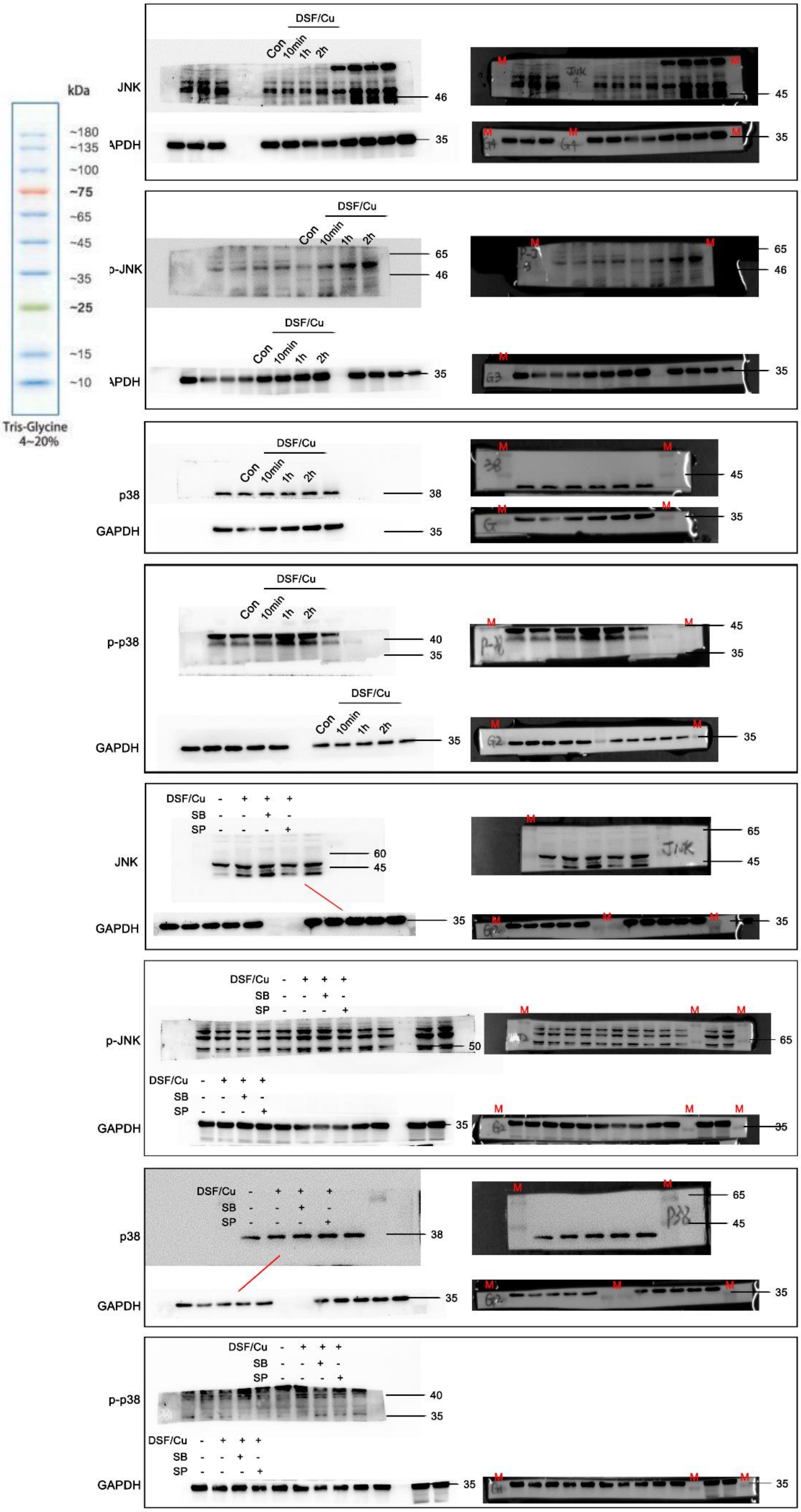
kDa
~180
~135
~100
~75
~65
~45
~35
~25
~15
~10
Tris-Glycine
4~20%
DSF/Cu
Con
10min
1h
2h
JNK
GAPDH
p-JNK
p38
p-p38
SB
SP
M
46
45
35
65
60
50
40
38

# Figure 5

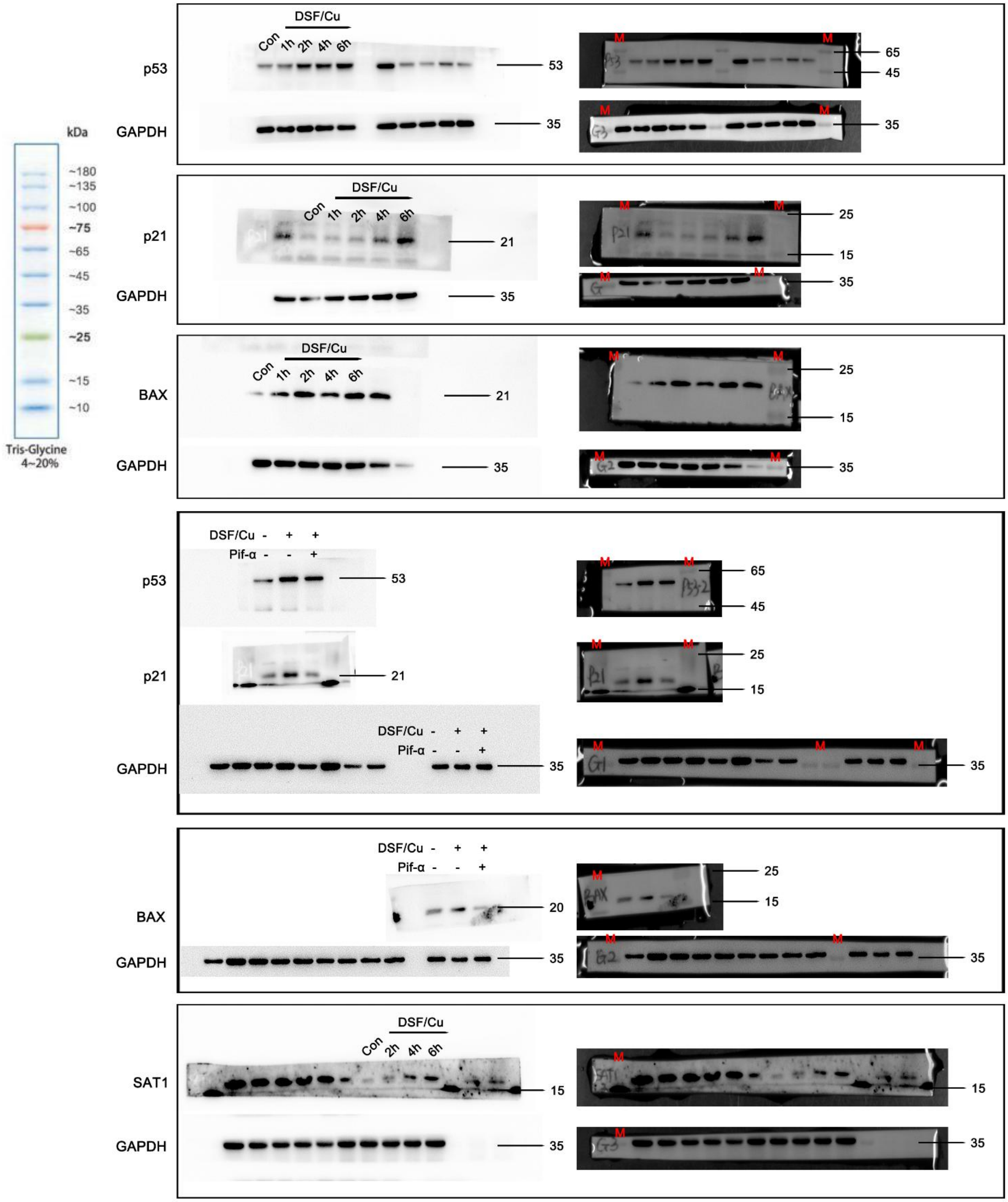

kDa
~180
~135
~100
~75
~65
~45
~35
~25
~15
~10
Tris-Glycine
4~20%
DSF/Cu
Con
1h
2h
4h
6h
p53
GAPDH
p21
BAX
SAT1
Pif-α
M
65
53
45
35
25
21
20
15

# Figure 6

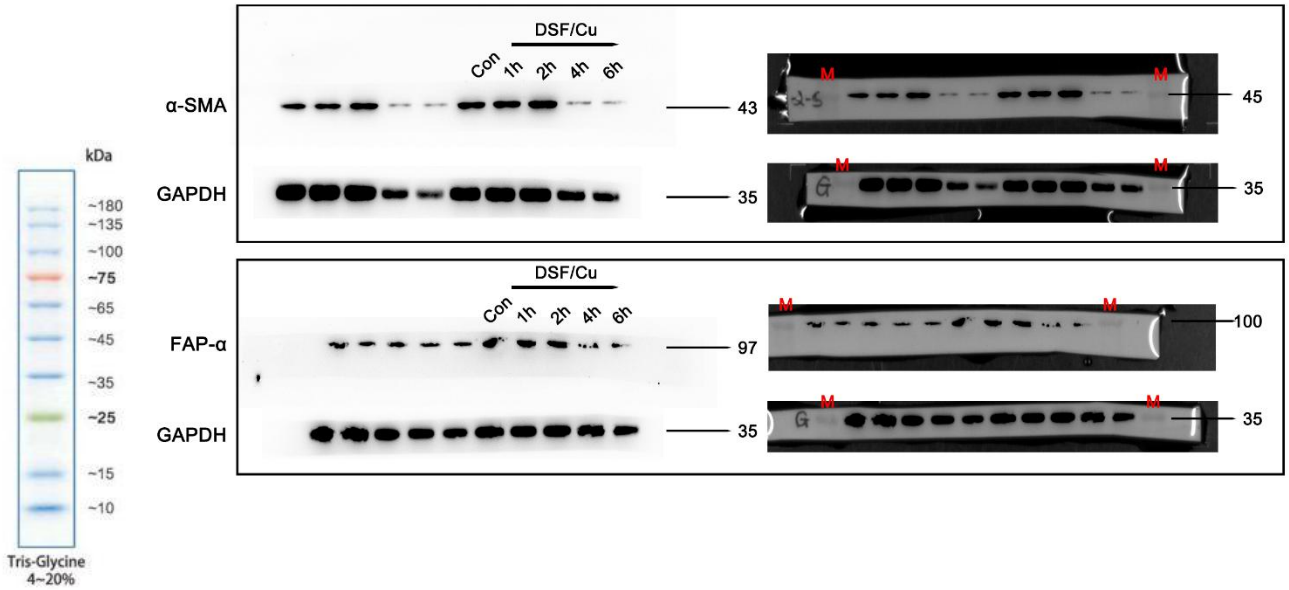

kDa
~180
~135
~100
~75
~65
~45
~35
~25
~15
~10
Tris-Glycine
4~20%
DSF/Cu
Con
1h
2h
4h
6h
α-SMA
43
GAPDH
35
M
45
35
FAP-α
97
100

**Figure 7**

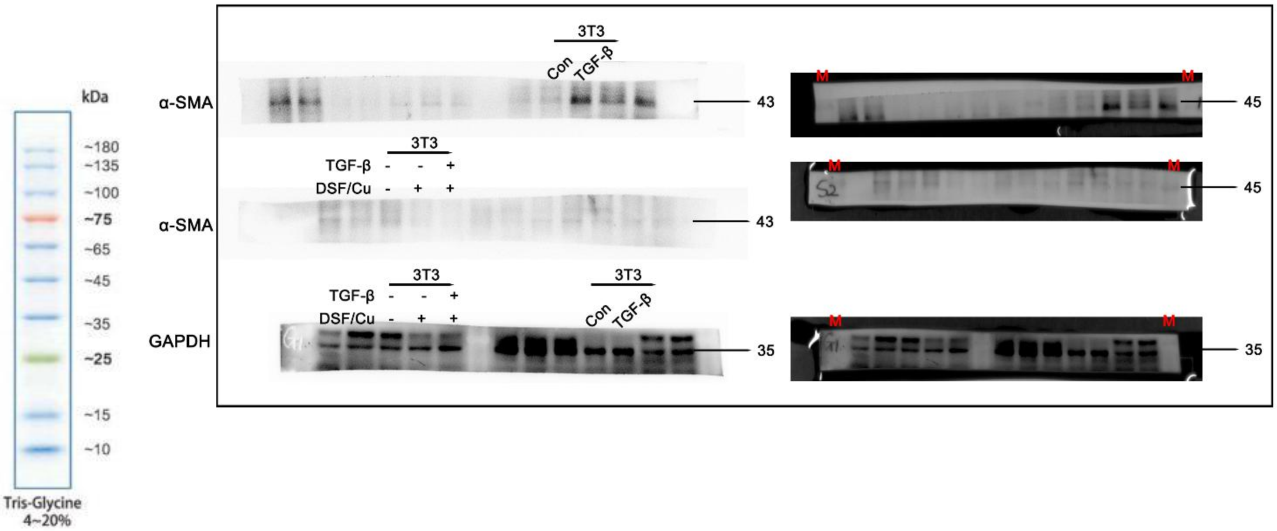
kDa
~180
~135
~100
~75
~65
~45
~35
~25
~15
~10
Tris-Glycine
4~20%
α-SMA
3T3
Con
TGF-β
43
TGF-β - - +
DSF/Cu - + +
α-SMA
43
GAPDH
35
M
45
M
45
M
35

# Figure S5

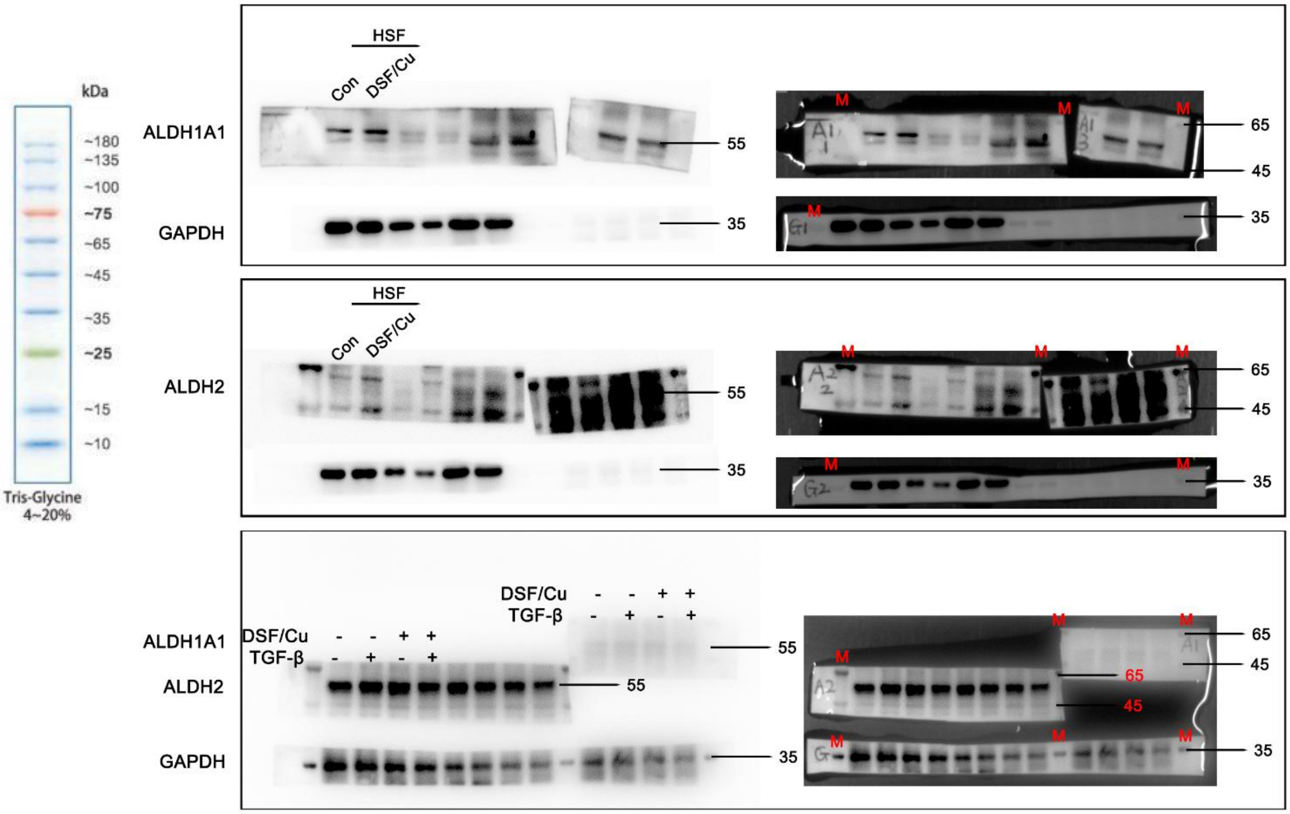

kDa
~180
~135
~100
~75
~65
~45
~35
~25
~15
~10
Tris-Glycine
4~20%
HSF
Con
DSF/Cu
ALDH1A1
GAPDH
55
35
65
45
M
ALDH2
DSF/Cu
TGF-β
