# Supplementary material for: Disulfiram/Copper Induces Antitumor Activity against Both Nasopharyngeal Cancer Cells and Cancer-Associated Fibroblasts through ROS/MAPK and Ferroptosis Pathways
Source: Cancers (Basel). 2020 Jan 6;12(1):138. doi: 10.3390/cancers12010138 (PMC7017005; doi:10.3390/cancers12010138)
Supplement: Supplementary file 1 [file cancers-12-00138-s001.zip › Supplementary.pdf]

Supplementary Data

Disulfiram/copper induces antitumor activity against both nasopharyngeal cancer cells and cancer-associated fibroblasts through ROS/MAPK and ferroptosis pathways

Yiqiu Li[1], Fangfang Chen[1], Jun Chen[1], Siocheong Chan[1], Yi He[2], Wanli Liu[2*], Ge Zhang[1*]

[1]Department of Microbial and Biochemical Pharmacy, School of Pharmaceutical Sciences, Sun Yat-sen University, Guangzhou, China

[2] State Key Laboratory of Oncology in South China, Collaborative Innovation Center for Cancer Medicine, Guangdong Key Laboratory of Nasopharyngeal Carcinoma Diagnosis and Therapy, Sun Yat-sen University Cancer Center, Guangzhou, P.R. China

[*]**Correspondence to:** Wanli Liu and Ge Zhang

Wanli Liu: State Key Laboratory of Oncology in South China, Collaborative Innovation Center for Cancer Medicine, Guangdong Key Laboratory of Nasopharyngeal Carcinoma Diagnosis and Therapy, Sun Yat-sen University Cancer Center, Guangzhou 510060, P. R. China. Tel: 86-20-8734-3199; Fax: 86-20-8734-3199; E-mail: liuwl@sysucc.org.cn

Ge Zhang: Department of Microbial and Biochemical Pharmacy, School of Pharmaceutical Sciences, Sun Yat-sen University, No.132 Waihuandong Road, University Town, Guangzhou 510006, China. Tel: 86-20-39943021; Fax: 86-20-39943021; E-mail: zhangge@mail.sysu.edu.cn

List of Contents:

Supplementary Figure

1. **Fig. S1:** DSF reduces the viability of nasopharyngeal carcinoma cells in a Cu-dependent manner**.**
2. **Fig. S2:** The cytotoxic effect of DSF/Cu on 5-8F is irreversible.
3. **Fig. S3:** DSF/Cu induces cytotoxicity via targeting the MAPK signaling pathway in 5-8F cells.
4. **Fig. S4:** DSF/Cu regulates the mRNA expressions of the p53 signaling and ferroptosis pathways in 5-8F cells.
5. **Fig. S5:** The expressions of ALDH1/2 in fibroblasts.
6. **Fig. S6:** CDDP/DSF/Cu significantly reduces the cell viability of nasopharyngeal carcinoma cells.
7. **Fig. S7:** H&E staining of the liver, lung, and kidney sections from mice treated with indicated drugs and PBS controls.
8. **Fig. S8:** DSF/Cu significantly reduces the viability of non-tumor cells.
9. **Fig. S9:** The concentration of copper is upregulated in liver after treated DSF/Cu in mice.

Supplementary Table

1. **Table S1:** The sequences of primers for the qRT-PCR analysis.
2. **Table S2:** Biochemical indices in control and DSF/Cu- treated mice.
3. **Table S3:** Blood routine indices in control and DSF/Cu- treated mice.

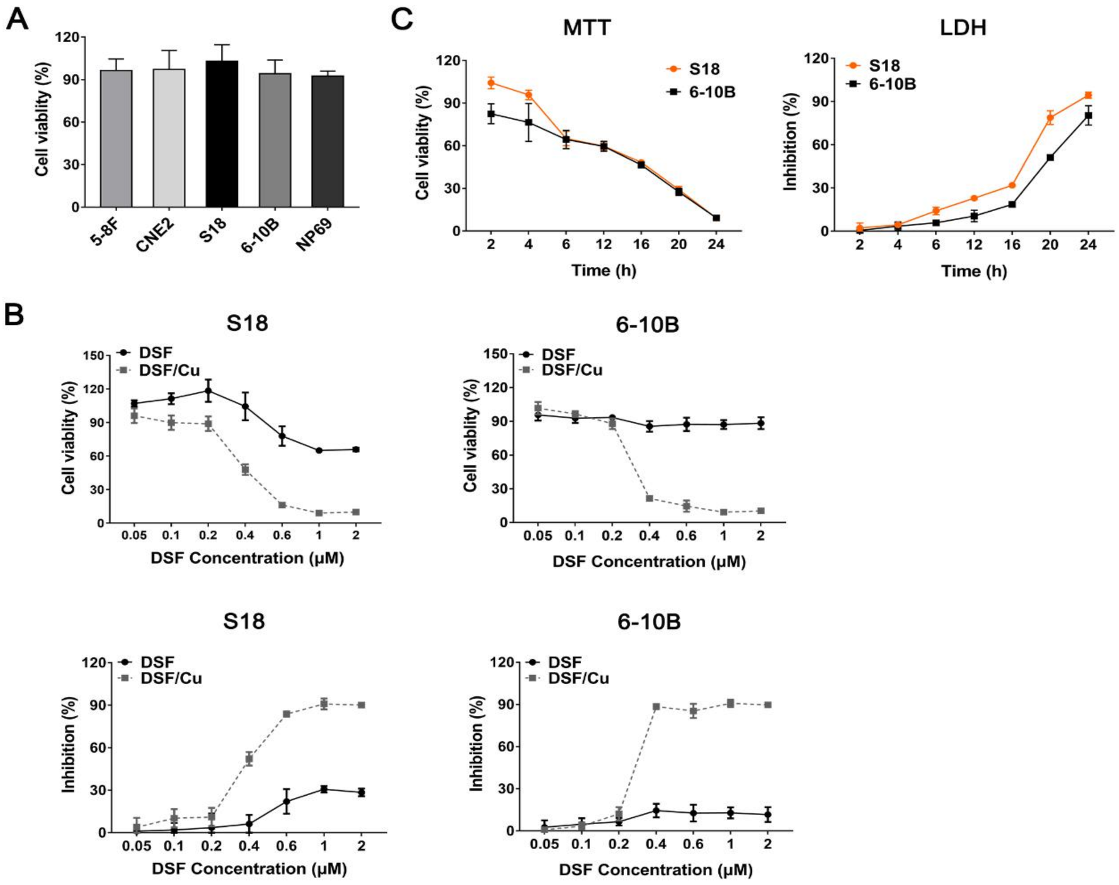

Fig. S1 DSF reduces the viability of nasopharyngeal carcinoma cells in a Cu-dependent manner. (A) The four NPC cells and the nonmalignant nasopharyngeal epithelial cells NP69-SV40T were treated with 1 μM Cu for 24 h. The cytotoxic effects were determined by MTT assay. **(B)** The NPC cells S18, 6-10B were exposed to the indicated concentration of DSF or with 1 μM Cu for 24 h, the cytotoxic effects were determined by MTT assay (above) and LDH assay (below). **(C)** The NPC cells S18, 6-10B were exposed to DSF/Cu (1 μM/ 1 μM) for indicated time, the cytotoxic effects were determined by MTT and LDH assay.

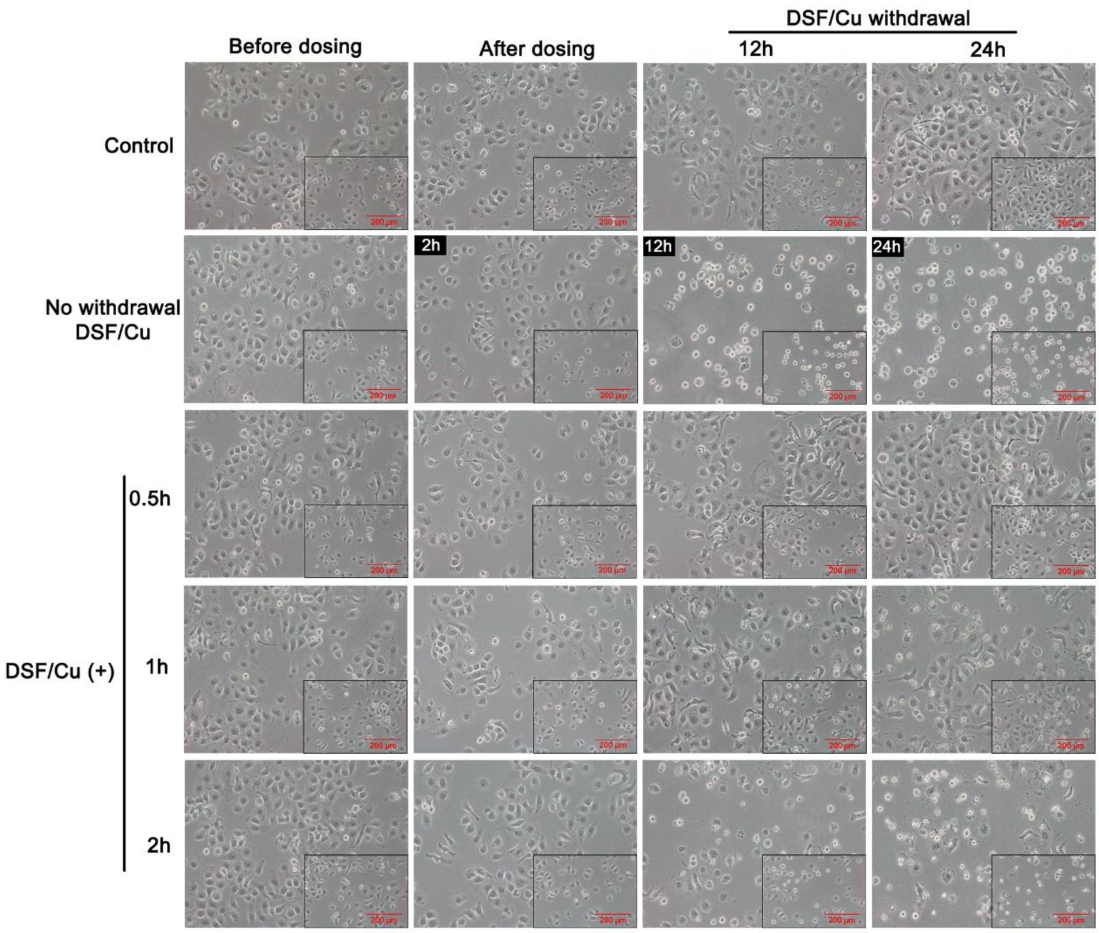

Fig. S2 The cytotoxic effect of DSF/Cu on 5-8F is irreversible. Morphological changes of NPC cells 5-8F after exposing to DSF/Cu (1 μM/ 1 μM) for indicated time and the cells were cultured for 12 h or 24 h after DSF/Cu withdrawal. Scale bar: 200 μm.

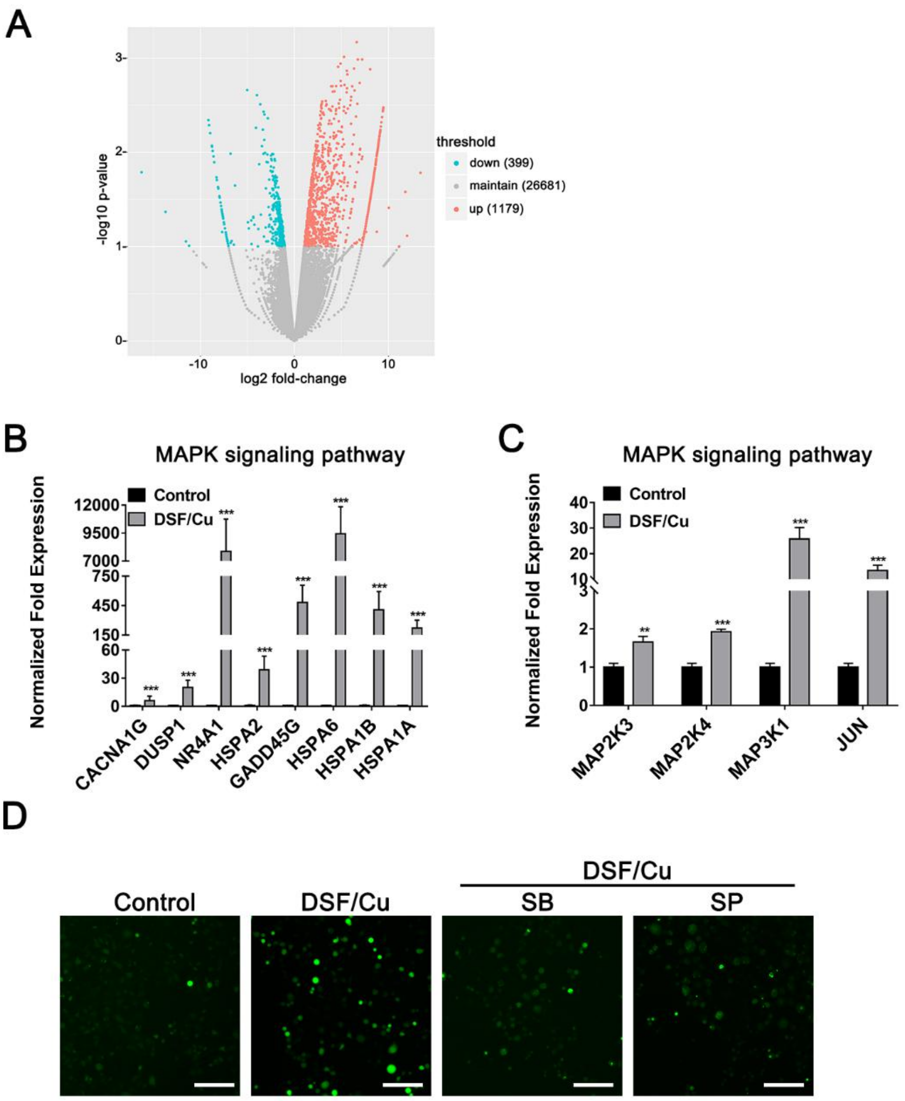

Fig. S3 DSF/Cu induces cytotoxicity via targeting the MAPK signaling pathway in 5-8F cells. (A) Gene expression volcanic map showed differential expressed genes in 5-8F after being exposed to DSF/Cu (1 μM/ 1 μM) for 4 h. **(B, C)** The mRNA expressions of several related genes and key genes in the MAPK signaling pathway, 5-8F cells were treated with DSF/Cu (1 μM/ 1 μM) for 5 h and measured by RT-qPCR. Data are shown as means ± SD. **P* < 0.05, ** *P* < 0.01, *** *P* < 0.001 vs control group, n = 3. **(D)** Representative images of 5-8F cells treated with DSF/Cu, DSF/Cu plus the p38 inhibitor SB (10 μM) or JNK inhibitor SP (10 μM) for 5 h from High

Content screening (HCS) assay. The inhibitors were pretreated 12 h before DSF/Cu was added. The images with magnification at 10 × are shown here, scale bar: 100 μm.

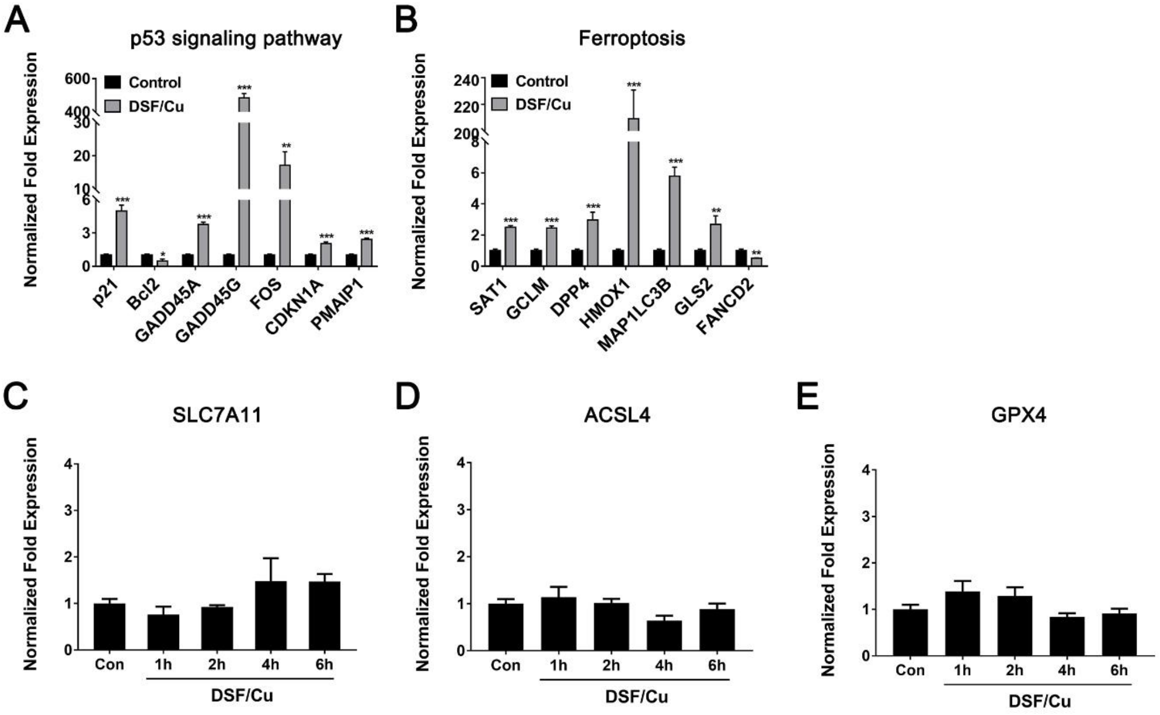

Fig. S4 DSF/Cu regulates the mRNA expressions of the p53 signaling and ferroptosis pathways in 5-8F cells. 5-8F cells were treated with DSF/Cu (1 μM/ 1 μM) for 5 h. DMSO was used as a control. **(A)** p53 signaling pathway. **(B)** Ferroptosis. **(C)** SLC7A11. **(D)** ACSL4. **(E)** GPX4. Data are shown as means ± SD. **P* < 0.05, ** *P* < 0.01, *** *P* < 0.001 vs control group, n = 3.

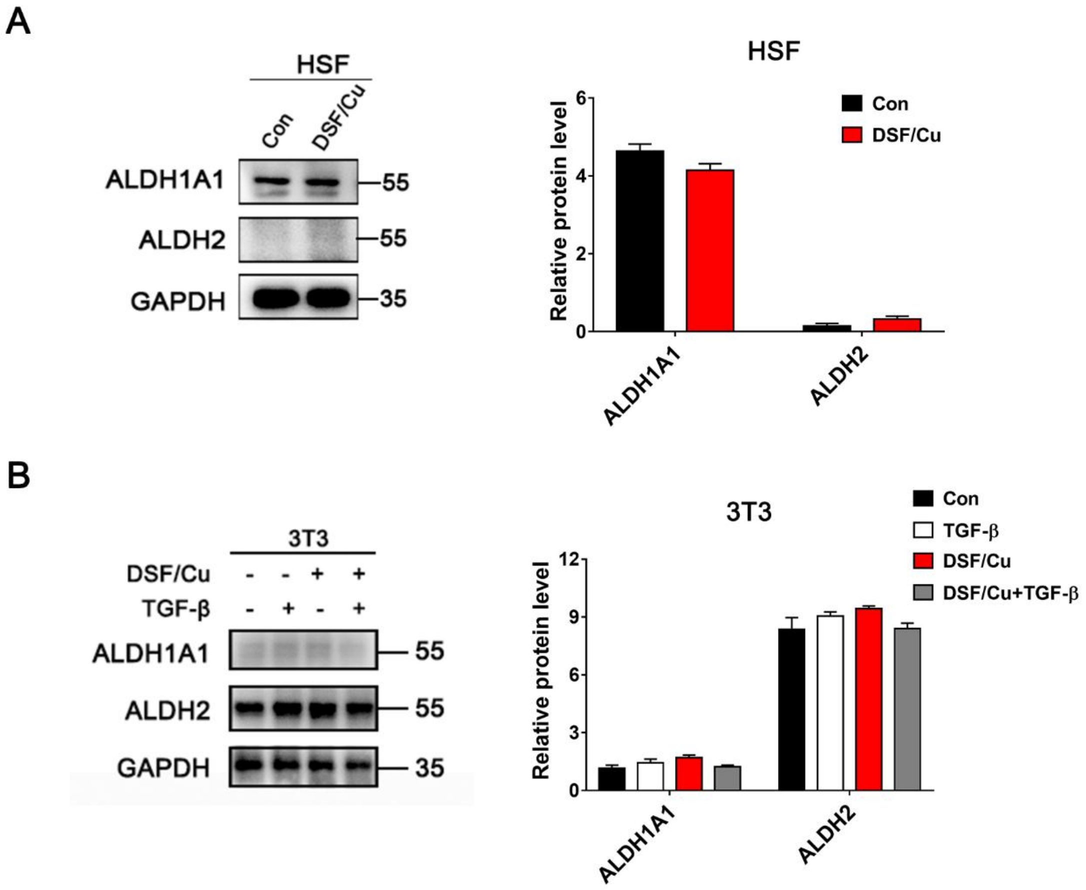

Fig. S5 The expressions of ALDH1/2 in fibroblasts. **(A)** ALDH1A1 and ALDH2 protein were detected by Western blot in HSF cells. HSF cells were exposed to DSF/Cu (1 μM/ 1 μM) for 6 h. **(B)** ALDH1A1 and ALDH2 protein were detected by Western blot in 3T3 cells. 3T3 cells were exposed to DSF/Cu (1 μM/ 1 μM) for 6 h. TGF-β1 (20 ng/mL) was pretreated for 48 h. Data are shown as means ± SD, n = 3.

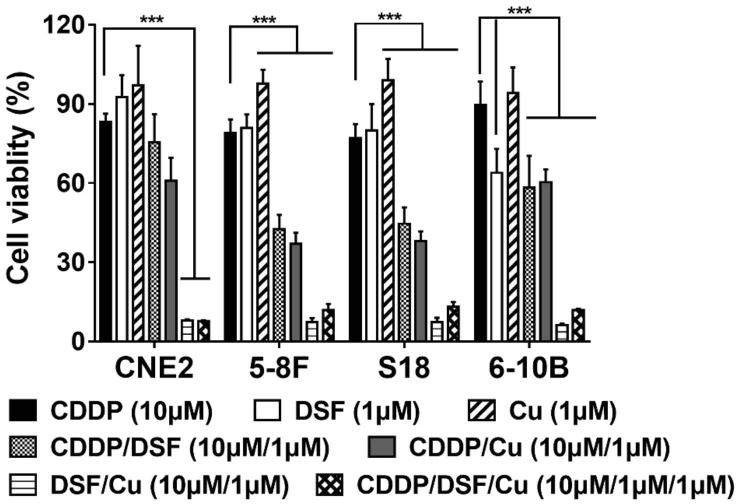

Fig. S6 CDDP/DSF/Cu significantly reduces the viability of nasopharyngeal carcinoma cells. CNE2, 5-8F, S18 and 6-10B were exposed to 10 μM CDDP, 1 μM DSF and 1 μM Cu for 24 h respectively, the inhibition effects were determined by MTT assay. Data are shown as means ± SD. *$P < 0.05$, ** $P < 0.01$, *** $P < 0.001$, n = 3.

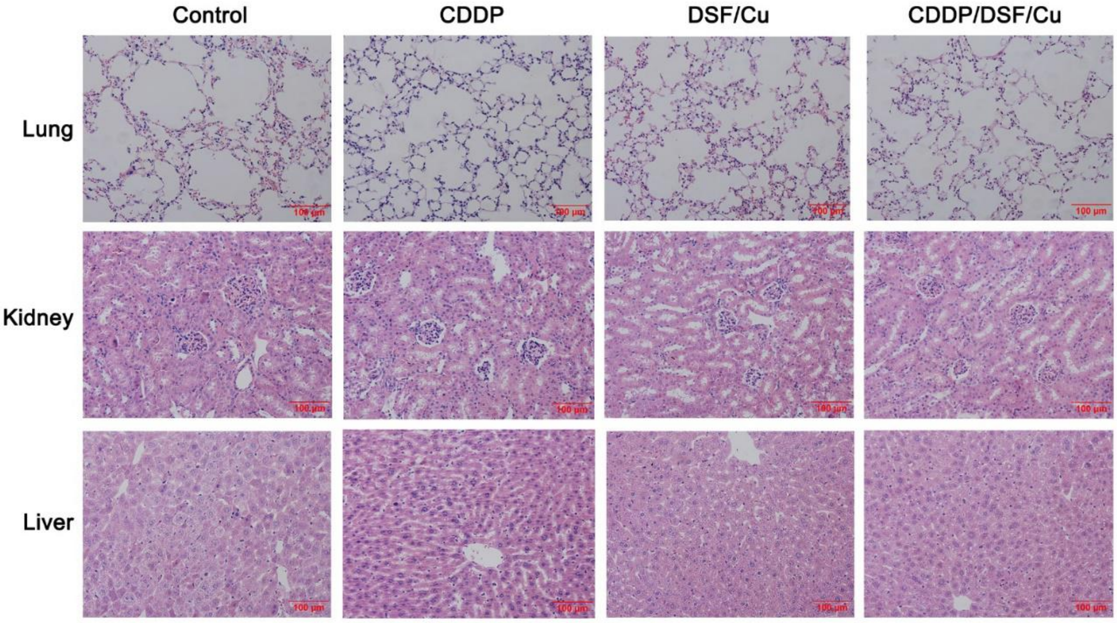

Fig. S7 H&E staining of the liver, lung, and kidney sections from mice treated with indicated drugs and PBS controls. 20 BALB/c nude male mice were randomly assigned to four groups (n = 5 per group) after the tumor grew to 150-200 mm^3 on average, each group was treated with the indicated drugs (i.p. PBS, CDDP and i.g. DSF, Cu, CDDP, PBS) and the mice were sacrificed after 13 days treatment. Scale bar: 100 μm.

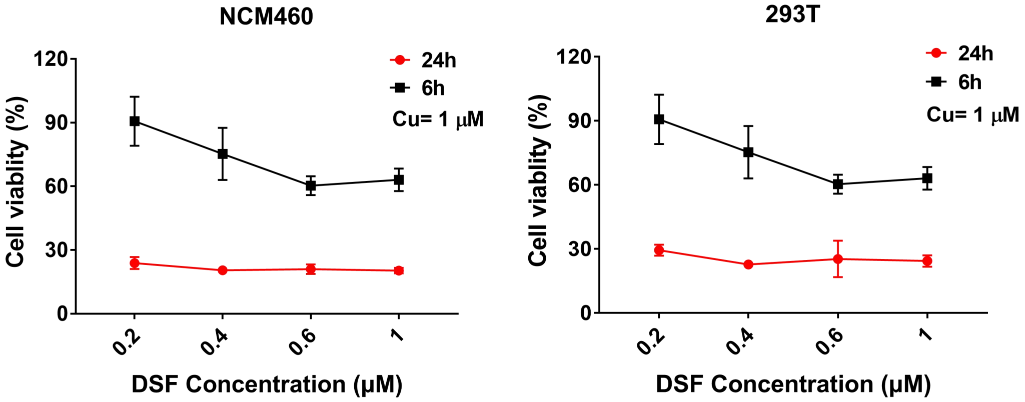

Fig. S8 DSF/Cu significantly reduces the viability of non-tumor cells. NCM460 and 293T cells were exposed to the indicated concentration of DSF with 1 μM Cu for 6 h or 24 h, the cytotoxic effects were determined by MTT assay.

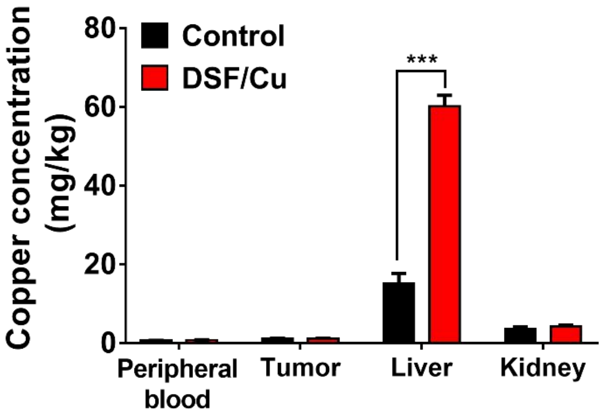

Fig. S9 The concentration of copper is upregulated in liver after treated DSF/Cu in mice. Data are shown as means ± SD. *$P < 0.05$, ** $P < 0.01$, *** $P < 0.001$, n = 3.

Table S1: The sequences of primers for the qRT-PCR analysis

Gene	Primer sequence
ALDH1A1	F:5'- GCCAGGTAGAAGAAGGAGATAAGGAGG -3'
	R:5'- TATAATAGTCGCCCCCTCTCGGAAG -3'
ALDH2	F:5'- ATCCCCATTGACGGAGACTTC -3'
	R:5'- CACAACCACGTTTCCAGTTGC -3'
CACNA1G	F:5'- AGAAGGAAAGCCCAGTGCAA -3'
	R:5'- CGGAAACCAAAGGCCACAAG -3'
DUSP1	F:5'- ACCACCACCGTGTTCAACTT -3'
	R:5'- CTCAAGGAGCATGGAGTCCC -3'
NR4A1	F:5'- GCAAGTGGGCGGAGAAGAT -3'
	R:5'- CCTCGCCTGGCTTAGACCT -3'
HSPA2	F:5'- GCACAAGAAGGACATTGGGC -3'
	R:5'- GCGCGTGATGGACGTATAGA -3'
GADD45G	F:5'- CATCGACATAGTGCGCGTG -3'
	R:5'- TCGAAATGAGGATGCAGTGC -3'
HSPA6	F:5'- GATGTGTCGGTTCTCTCCATTG -3'
	R:5'- CTTCCATGAAGTGGTTCACGA -3'
HSPA1B	F:5'- AGCTGGAGCAGGTGTGTAAC -3'
	R:5'- TCCTCAATGGTAGGGCCTGA -3'
HSPA1A	F:5'- AGCTGGAGCAGGTGTGTAAC -3'
	R:5'- TACCTCCTCAATGGTGGGGC -3'
MAP2K3	F:5'- TGGCATCAGTGGCTACTTGG -3'
	R:5'- TCAGGCACTGAGCAGTGAAG -3'
MAP2K4	F:5'- TGAAAAGGCACAAAGTAAACGCA -3'
	R:5'- CCCAGTGTTGTTCAGGGGAG -3'
MAP3K1	F:5'- AAGTGCGGAGTGTGGAGCTG -3'
	R:5'- CGACTCGGTAAGGTGGGCG -3'
JUN	F:5'- GTGCCGAAAAAGGAAGCTGG -3'
	R:5'- CTGCGTTAGCATGAGTTGGC -3'
p21	F:5'- GACTCTCAGGGTCGAAAACGG -3'
	R:5'- GCGGATTAGGGCTTCCTCTT -3'
Bcl2	F:5'- TCCATTATAAGCTGTCACAG -3'
	R:5'- GAAGAGTTCCTCCACCAC -3'
GADD45A	F:5'- CCACATTCATCTCAATGGAAG -3'

	R:5’- GGGAGATTAATCACTGGAACC -3’
FOS	F:5’- TACTACCACTCACCCGCAGA -3’
	R:5’- GGCCTCCTGTCATGGTCTTC -3’
CDKN1A	F:5’- GCCCAGTGGACAGCGAGCAG -3’
	R:5’- GCCGGCGTTTGGAGTGGTAGA -3’
PMAIP1	F:5’- GCAAGAACGCTCAACCGAG -3’
	R:5’- TCTGCCGGAAGTTCAGTTTGT -3’
SAT1	F:5’- AGAGCACCCCTTTTACCACTG -3’
	R:5’- TGGCAAAACCAACAATGCTGT -3’
GCLM	F:5’- GGAACCTGCTGAACTGGGG -3’
	R:5’- GAACAGGCCATGTCAACTGC -3’
DPP4	F:5’- GCACGGCAACACATTGAA -3’
	R:5’- TGAGGTTCTGAAGGCCTAAATC -3’
HMOX1	F:5’- GGCAGAGAATGCTGAGTTCA -3’
	R:5’- CCACATAGATGTGGTACAGG -3’
MAP1LC3B	F:5’- GGCCTTCTTCCTGTTGGTGA -3’
	R:5’- TCTCCTGGGAGGCATAGACC -3’
GLS2	F:5’- TTCAGCAATGCCACGGAAGG -3’
	R:5’- ATCATGTCCACCCCCTTAGGA -3’
FANCD2	F:5’- CCCAAGAGAGAGCCAACCTG -3’
	R:5’- GGTATGCCCAACCCATTCCA -3’
ALOX15	F:5’- AGCCTGATGGGAAACTCTTG -3’
	R:5’- AGGTGGTGGGGATCCTGT -3’
Ptgs2	F:5’- GGCCATGGGGTGGACTTAAA -3’
	R:5’- CCCCACAGCAAACCGTAGAT -3’
SLC7A11	F:5’- ATGCAGTGGCAGTGACCTTT -3’
	R:5’- GGCAACAAAGATCGGAACTG -3’
ACSL4	F:5’- TCAGCAACAGCAAACAGACC -3’
	R:5’- CCAAGAGCAAGGAAGGTAAAAAG -3’
GPX4	F:5’- TTCCCGTGTAACCAGTTCG -3’
	R:5’- CGGCGAACTCTTTGATCTCT -3’
GAPDH	F:5’- GACTCATGACCACAGTCCATGC -3’
	R:5’- AGAGGCAGGGATGATGTTCTG -3’

Table S2

Biochemical indices in mice treated with DSF/Cu (150 mg/kg / 2 mg/kg) with or without CDDP (5 mg/kg)

Groups	ALT (U/L)	AST (U/L)	ALB (g/L)	URE (mmol/L)	CRE (mmol/L)	TBIL (μmol/L)
Control	41.5±14.50	98.6±16.55	29.3±3.26	9.7±0.57	10.5±1.13	0.1±0.28
DSF/Cu	31.0±7.28	128.9±77.86	33.6±1.41*	9.6±0	26.5±22.49	1.5±0.28*
CDDP/DSF/Cu	32.4±3.68	132.0±9.62	27.4±0.57	34.6±22.63	15.9±3.25	1.8±0.57*

Note: compared with control group, *$P < 0.05$, n = 3.

Table S3

Blood routine indices in mice treated with DSF/Cu (150 mg/kg / 2 mg/kg) with or without CDDP (5 mg/kg)

Groups	WBC/ (10^9/L$^/$)	RBC/ (10^{12}/L$^/$)	PLT/ (10^9//L$^/$)
Control	3.12±2.51	10.47±0.15	1528.0±215.31
DSF/Cu	6.68±3.49	9.28±1.10	1390.5±234.05
CDDP/DSF/Cu	5.55±0.84	7.12±1.34	1254.3±386.52

Note: n = 3.
